# Research progress on the role of the Wnt signaling pathway in pituitary adenoma

**DOI:** 10.3389/fendo.2023.1216817

**Published:** 2023-09-14

**Authors:** Wencai Wang, Luyao Ma, Yongqiang Zhao, Menghao Liu, Wei Ye, Xianfeng Li

**Affiliations:** Department of Neurosurgery, The Second Affiliated Hospital of Harbin Medical University, Harbin, China

**Keywords:** PA, Wnt signaling pathway, Wnt pathway inhibitors, targeted drugs, non-coding RNA, TME

## Abstract

Pituitary adenoma (PA) is the third most common central nervous system tumor originating from the anterior pituitary, but its pathogenesis remains unclear. The Wnt signaling pathway is a conserved pathway involved in cell proliferation, Self-renewal of stem cells, and cell differentiation. It is related to the occurrence of various tumors, including PA. This article reviews the latest developments in Wnt pathway inhibitors and pathway-targeted drugs. It discusses the possibility of combining Wnt pathway inhibitors with immunotherapy to provide a theoretical basis for the combined treatment of PA.

## Introduction

PAs are the most common tumor in the sellar region, accounting for 10–15% of the total intracranial tumors (1) PAs arise from the adenohypophyseal cells of the anterior pituitary lobe and are benign tumors (2). There are general symptoms associated with PAs, such as hormone overproduction and compression of the optic chiasm and pituitary gland (3, 4). According to functional classification, PAs can be divided into active and inactive PAs. Functional PAs can be classified as prolactinoma, growth hormone adenoma, adrenocorticotropic hormone adenoma, thyrotropin-secreting adenoma, gonadotropin-secreting adenoma, mixed adenoma, unclassified adenoma (5). Despite recent progress in molecular genetics, the pathogenesis of PAs still needs to be fully understood since confirmed that it originated from monoclonal cells in 1990 (6). Recent researches indicate that the pathogenesis of PAs may be influenced by cell type specificity, gene mutation, and epigenetic changes (7, 8). Also, abnormal activation of the wnt pathway has been found to play a crucial role in developing PAs.

## Wnt signaling pathway

The Wnt signaling pathway is an ancient evolutionarily conserved pathway mediated by Wnt proteins. It involves multiple events during embryonic development and tissue homeostasis, including cell multiplication, stem cell self-renewal, and cell differentiation (9, 10).

Historically, the Wnt signaling pathway has been divided into two broad categories: canonical and noncanonical (10, 11). Canonical Wnt signaling is activated when Wnt proteins increase or Wnt signaling inhibitors decrease. When Wnt proteins bind to their specific cell membrane receptors, they activate FZD and LRP5/6 (12). It then activates DVL and inhibits the multiprotein activity of GSK3β, Axin1/Axin2, APC, and CK1 (13). Blocks β-catenin phosphorylation, resulting in cytoplasmic accumulation of β-catenin. Finally, the β-catenin accumulated in the cytoplasm is transported to the nucleus, where it interacts with LEF and TC, targeting downstream target genes CyclinD1, c-myc, MMPs, Etc., triggering transcriptional activation and promoting cell proliferation or migration (14, 15).

The atypical pathway is β-catenin independent and primarily activated by Wnt5a. It can be divided into two pathways, Wnt/Ca2+ and PCP, whose primary function is to regulate cell polarity and migration (16). The Wnt5a protein binds to the FZD protein, activates DVL, and triggers PLC to release calcium ions. Calcium-sensitive enzymes such as PKC, CaMkII, and calcium-sensitive calcineurin are activated. Ultimately, the activation of NFAT leads to transcriptional activation, cytoskeletal rearrangement, and cell adhesion and migration regulation (17–21). In the PCP pathway, the Wnt5a protein binds to FZD receptors, activates DVL, sparks Rho-associated kinases and Rac/JNK signaling, and stimulates intracellular actin polymerization. It ultimately regulates cell polarity and migration (22–24).

**Figure 1:**
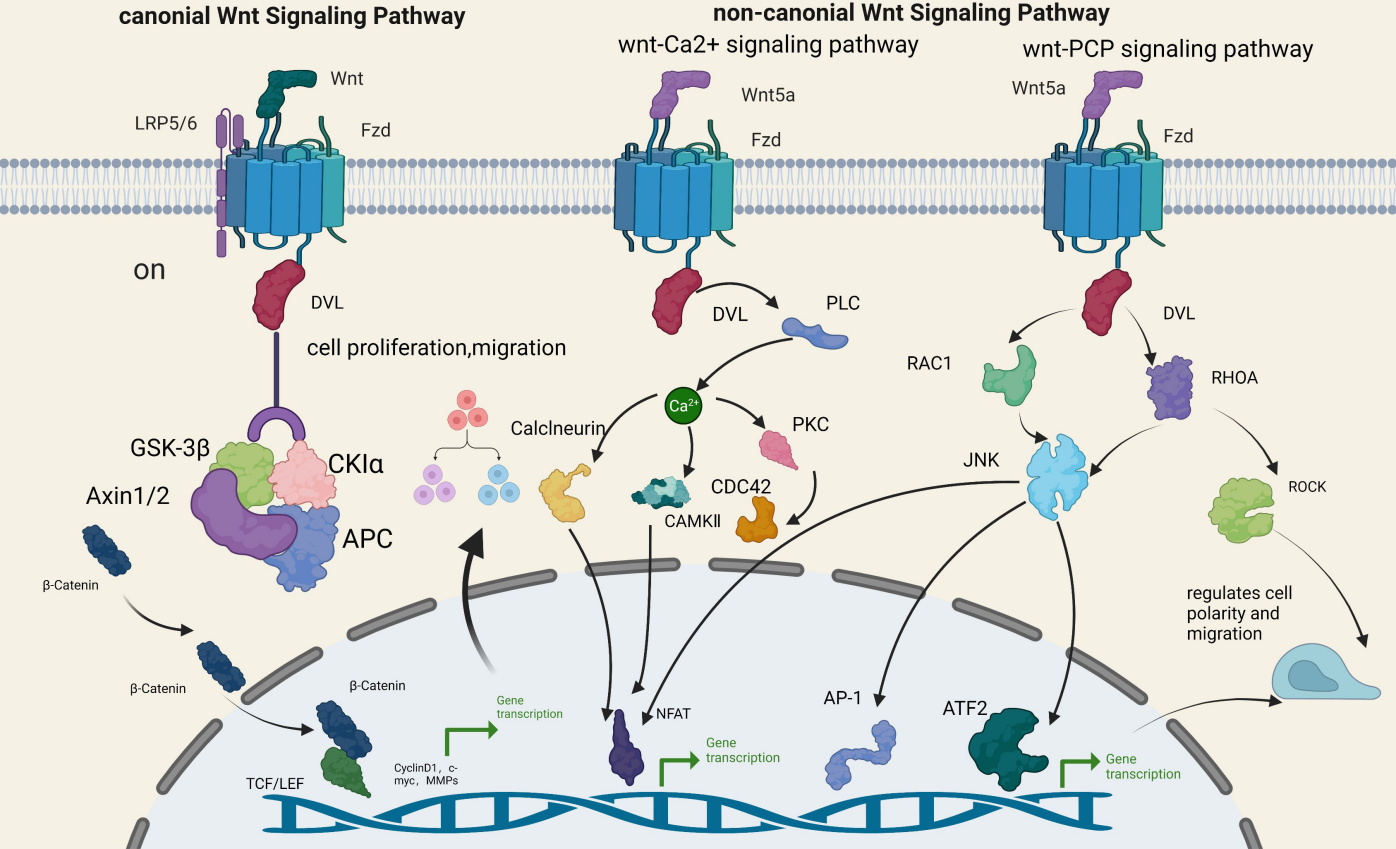
Wnt signaling pathway.

Research has demonstrated that abnormal activation of the Wnt pathway is relevant to the progression and pathogenesis of many neoplasms, including breast carcinoma (25), melanoma (26), Barrett’s esophagus (27), and colorectal cancer (28). These two signaling pathways often form a network of overlapping signals and mutual regulation, involving the recrudesce and progression of multiple illnesses (**Figure 1**).

## Wnt signaling pathway and PA

As early as 2005, Moreno et al. found that altered expression of SFRP1, TLE2, PITX2, NOTCH3, and DLK1 was observed in NFPA. The Wnt pathway may be crucial to the progression of NFPA (29). The Wnt4 molecule is a secreted glycoprotein involved in the multiplication and differentiation of normal and malignant cells. Although the expression of Wnt4 in various neoplasms has been extensively studied, its function in PAs remains unknown. In 2011, Gilles et al. showed that Wnt4 affects informal pituitary signals by restraining Ca2+ oscillations in GH3 cells for the first time, but the downstream effects are uncertain. It also suggests that Wnt4 is participated in adult tissue plasticity and may be related to β-catenin (30). In 2014, Weiping Li et al. discovered that the Wnt4 pathway was dysregulated in most PAs, and its overactivation could inhibit the invasion of PAs (31).

As the core protein of the canonical Wnt pathway, β-catenin is a downstream protein of Wnt4. It is crucial in promoting cellular proliferation and invasion in the PAs. In 2002, Howng et al. discovered that mutations in β-catenin, E-cadherin, and Wnt signaling pathways have participated in the progression of cerebral neoplasms (32). In 2014, Weiping Li and others found that Wnt4 and β-catenin were also overexpressed in most PAs in addition to ACTHPA (31). Furthermore, β-catenin is inversely correlated with the invasiveness of PAs. In 2015, Zhao et al. discovered that the stable β-catenin gene knockout PAs’ cell line was transfected with a plasmid. Then the cellular multiplication and invasion ability were detected by the CCK-8 kit and Transwell method. It was found that β-catenin could regulate AKT and STAT3. The expression of MMP-2/9, cyclin D1, and CDK4 boosted the multiplication and invasiveness of PAs (33).

Other downstream signaling targets, such as c-myc, have also been shown to be essential for forming PAs. C-myc may facilitate tumor growth, cell transformation, and evolution. Nevertheless, c-myc did not affect the recurrence of functional tumors (34). In 2017, Liu et al. found that β-catenin and c-myc are helpful immunohistochemical biomarkers for detecting invasive NFPA. However, they need to be validated in many samples before they can be used to predict the recurrence of NFPA (35).

In addition, pituitary homeobox 2, one of the downstream signaling targets of the Wnt pathway, also plays a significant part in pituitary formation. In 2011, Acunzo et al. found that the Wnt pathway-induced PITX2 gene may participate in the emergence of gonadotropin-secreting adenoma and NFPA through anti-apoptosis (36). In 2013, Lee et al. demonstrated for the first time that multidrug resistance p-glycoprotein, as a target gene of PITX2, can boost multidrug resistance and cellular endurance of cancer cells (37). Therefore, comprehending the function of the Wnt pathway in PAs could be further enriched by examining PITX2.

However, PAs often shares multiple signaling pathways with other tumors rather than being induced by wnt signaling alone. As the study by Hosseinkhan et al., in NFPA, Wnt, MAPK, TGF-b, Hippo, VEGF, JAK-STAT, PI3K-Akt, ErbB, TGF-b, and Ras signaling pathways are intertwined and jointly participate in the formation of tumors (38). Crosstalk between the Wnt pathway and other pathways is common in many tumors, including melanoma (39). Likewise, it was recently discovered in PAs. In 2019, SOX2 was shown to promote the cell proliferation of PAs via intermediating crosstalk between Wnt and Shh pathways (40).

## Inhibitors of Wnt signaling and PAs

Conventionally, inhibitors of Wnt signaling can be divided into two categories. The first class includes the SFRP family, WIF1, and Cerberus (41). They could theoretically block canonical or noncanonical pathways via directly binding to Wnt ligands and inhibiting their association with FZD receptor complexes (42). The second group mainly includes the DKK families. They block the canonical Wnt pathway via binding to LRP5 or LRP6 coreceptors (43, 44).

The SFRP family, located at 8q12-11.1, participates in the competitive binding of Wnt proteins through the N-terminal CRD structure (homologous to the FZD receptor). It incorporates five members (SFRP1-SFRP5). Melkonyan et al. identified SFRP1, SFRP2, and SFRP5 in mouse embryonic cells (45). By contrast, Rattner et al. discovered SFRP3 and SFRP4 (46). SFRP, an antagonist in several cancers, is a downstream target of the Wnt pathway. It plays a vital part in PA’s wnt signaling. In 2015, Wu et al. first found that SFRP4 promoter methylation was increased in invasive PAs and that SFRP4 promoter methylation decreased SFRP4 expression (47). The following year, the team found that sFRP2 inhibits tumor growth and invasion by regulating the wnt signaling pathway. The study is the first to show that sFRP2 was negatively correlated with the aggression of NFPA (48). In 2018, Ren et al. found an association between low expression of SFRP2 and features of invasive adenomas, including more prominent size and invasiveness. Overexpression of SFRP2 decreased β-catenin and suppressed the Wnt pathway in ACTH adenoma cells, thereby reducing the production of ACTH (49). Therefore, sFRP4 and sFRP2 may play a tumor suppressor role in PAs, and their expression may serve as biomarkers of aggressiveness and prognosis in PAs.

WIF1, the first secretory antagonist found in the retina, is located at 12q14.3 and is a crucial regulator of Wnt signaling (50–52). Hypermethylation of CpG promoters is downregulated. It directly relates to Wnt protein binding and inhibits the receptor signaling complex. When Wnt protein increases and WIF1 decreases, Wnt signaling is activated. Then Dsh is activated, which inhibits the activity of the APC-axin-GSK3 complex, resulting in the blocking of the phosphorylation and degradation of β-catenin. Finally, it results in β-Catenin gathering stably in the cytoplasm. β-catenin also translocates to the nucleus and plays a role in activating the transcriptional activity of T cell factor/lymphocyte enhancer, inducing and activating downstream target genes CyclinD1, c-myc, MMPs, Etc. It promotes and participates in forming tumors that can grow or migrate. In 2018, Song et al. found that the downregulation of WIF1 and sFRP4 are inversely correlated with the aggressiveness of NFPA. The results showed that miRNA-137 inhibited the invasion of NFPA by affecting the methylation of the WIF1 promoter and down-regulating WIF1 in the Wnt signaling pathway (53). One month later, Zhu et al. discovered that the expression of TGF-b1 and WIF1 in NFPA is correlated with cell multiplication and relapse (54). In recent years, studies have found that during the development of cancer cells, the transcription of SLC20A1 can absorb phosphate from interstitial fluid for cell metabolism, Signal transduction, and nucleic acid synthesis. In 2019, Li et al. found that high levels of SLC20A1 could reduce the protein levels of SFRP4 and WIF1 by activating the Wnt pathway, then facilitating the multiplication, invasiveness, and recurrence of GH adenoma. SLC20A1 is positively associated with β-catenin and negatively associated with WIF1 (55). At the same time, Cheng established a clinical prediction model from 295 NFPA tumor samples and found that three proteins, CDKN2A/p16, WIF1, TGF-β, tumor age and volume, and two clinical features were significantly associated with the recurrence of NFPA (56). Soon, Lei et al. found that miR-137 upregulated WIF1 in the Wnt pathway and inhibited the nuclear translocation of β-catenin by affecting the promoter methylation of WIF1, thereby inhibiting the growth and invasion of PRL tumors (57). Therefore, WIF1, as the primary inhibitor of the Wnt pathway, widely regulates the growth, invasion, and proliferation of PAs and can be used as a promising biomarker of PAs.

The DKK family is located at 10q11 and is mainly related to the atypical Wnt pathway by binding LRP5/6 and may simultaneously affect the canonical inhibitors of the Wnt pathway (43). DKK4 behaves like DKK1. However, the role of DKK2 in Wnt signaling remains controversial. Studies have shown that DKK2 could inhibit and activate the Wnt pathway under certain conditions (58). DKK3 (also known as DKKL1) is still considered an inhibitor of Wnt signaling, but its function must be fully comprehended (41). The clinical features of ACTH adenoma and acromegaly are usually disorders of bone metabolism, often accompanied by abnormal osteomalacia and osteoporosis. Research has confirmed that the Wnt pathway is essential in osteogenesis (59). In 2018, Belaya et al. found that acromegaly affects osteoblasts by significantly increasing the mRNA levels of DKK1 and wnt10B and altering the expression of miRNAs that participated in mesenchymal stem cellular binding (60). ACTH adenomas produce cortisol through the ACTH target gland axis, leading to osteoporosis in patients. The team also found that excess endogenous GC in humans inhibits osteogenesis by upregulating DKK1 and inactivating microRNAs involved in mesenchymal stem cell binding (61). Therefore, the Wnt pathway cannot be ignored for the secondary complications of osteoporosis and skeletal dysplasia in patients of PA. As a potent inhibitor of this signaling pathway, DKK also plays a crucial regulatory part. Therefore, further research on the Wnt pathway is significant for rationalizing treatment options and preventing subsequent complications in patients with PA.

Recently, many researchers have focused on non-coding RNAs, which also have inhibitory effects on Wnt signaling. In 2018, Song et al. miRNAs, including miRNA-137, were found to affect Wnt signaling by regulating WIF1 methylation (53). In 2019, Wang et al. showed that foxp1-induced CLRN1-AS1 ncRNA suppresses PRL tumors by blocking the miR-217/DKK1 axis to inactivate the Wnt pathway (62). In December of the same year, Lei et al. showed that miR-137 suppresses the growth and aggression of PRL tumors via inhibiting the Wnt pathway, upregulating WIF-1, and inhibiting β-catenin nuclear translocation (57). Currently, published studies have shown that many non-coding RNAs directly or indirectly inhibit the Wnt pathway, thereby inhibiting the development of PAs. In 2019, Shen et al. found that microRNA-543 downregulates Smad7, activates the Wnt pathway, boosts the invasion of PAs, and prevents apoptosis (63). In October of the same year, Zhang et al. PVT1 (lncRNA) was discovered to take a tumorigenic part in PA by activating the Wnt pathway (64). CircRNAs are one of three common ncRNAs that act as microRNA sponges to reverse the repression of target genes by disease-associated miRNAs and regulate disease-associated progression. CircRNAs involve the relapse and growth of various neoplasms, such as hepatic carcinoma (65), by promoting the Wnt signaling pathway. In 2020, Du et al. Through extensive analysis of circular RNAs, it was discovered that the involvement of hsa_circ_0001368 can noticeably promote the multiplication, aggressiveness, and GH secretion levels of GHPA cells. Database analysis found that the wnt pathway is one of the most enriched pathways for target genes (66). Therefore, circRNAs may participate in the development and pathogenesis of PAs by promoting wnt signaling. In 2022, Vetrivel et al. described the ACTH-independent expression of miR-1247-5p and miR-379-5p in CS for the first time. Then, they identified distinct adrenal miRNAs associated with CS subtypes and showed that miRNAs regulate Wnt signaling. Different genes may be individually involved in the pathology of specific subtypes of Cushing’s disease (67). Therefore, ncRNAs may not only act as inhibitors of the Wnt pathway to inhibit the tumorigenesis of PAs but also function in PAs as activators of the Wnt pathway. However, the specific mechanism of ncRNA’s dual role in PAs is unclear and needs further investigation.

According to previous studies, the abnormal expression of wnt pathway inhibitors may be related to PAs’ multiplication, aggressiveness, and typing. The research on Wnt pathway inhibitors will help to study the detailed mechanism of drugs targeting the Wnt pathway and treat PAs and their complications (**Table 1**).

**Table 1:** Related findings of Wnt pathway inhibitors in PA.

Wnt pathway inhibitor.		years.	Related findings.
SFRPs family.	SFRP4	2015	(47)
	sFRP2	2016	(48)
		2018	(49)
WIF1		2018	(53)
		2018	(54)
		2019	(55)
		2019	(56)
		2019	(57)
DKKs family	DKK1	2018	(60)
		2018	(61)
ncRNAs	miRNA-137(-)	2018	(53)
	lncRNA CLRN1-AS1(-)	2019	(62)
	miR-137(-)	2019	(57)
	microRNA-543(+)	2019	(63)
	PVT1 (+)	2019	(64)
	hsa_circ_0001368	2020	(66)
	miR-1247-5p, miR-379-5p	2022	(67)

## Drugs that target the Wnt pathway are used to treat PAs

Currently, prolactinomas are typically treated with drug therapy, whereas other types of PAs are often treated with surgery (68). If the symptoms can be treated or alleviated by targeting biological factors, it can bring great convenience to the clinical management of patients and reduce the need for surgery. Therefore, more research on non-surgical treatment is needed. In recent years, drug research targeting the wnt pathway has undoubtedly opened up a new direction for treating PAs.

In 2012, Bai et al. found that fulvestrant is a novel estrogen receptor antagonist that downregulates the expression of estrogen receptor α and Wnt4, upregulates the expression of WIF-1 and inhibits the cell proliferation of GH3 through estrogen receptor α and Wnt signaling pathways (69). The following year, the research team found through *in vitro* experiments that fulvestrant is closely related to the atypical Wnt signaling pathway. Furthermore, they also suggest that fulvestrant can be used as a therapeutic agent (70). In two 2014 papers, Cao and co-workers showed that fulvestrant could inhibit the cellular multiplication of PAs and the secretion of PRL via the Wnt pathway (71, 72). Fulvestrant’s main side effects are vasodilation and nausea (73). It is also hepatotoxic and can cause clinical symptoms such as jaundice and elevated transaminases (74). In 2019. Lei et al. found that decitabine increases WIF1 and promotes miR-137 targeting MITF. Moreover, it ultimately affects Wnt signaling, inhibiting the growth and invasion of prolactinoma (57). The most common side effects of decitabine were neutropenia, thrombocytopenia, and febrile neutropenia (75, 76).

By controlling the Wnt pathway, NEK2 can regulate the biological behavior of neoplasms, including liver cancer (77), glioblastoma (78), Etc. In 2021, Jian et al. confirmed that overexpression of NEK2 can dramatically boost the multiplication of PA cells by promoting the Wnt signaling pathway and reducing the ability of cells to have CAB sensitivity (79). Therefore, the antitumor effect of dopamine receptor agonists on PA can be enhanced by knocking down NEK2 and inhibiting wnt signaling. However, CAB should be used with particular caution in valvular heart disease and psychiatric disorders (80).

In addition, the antineoplastic drug TMZ was recently found effective against pituitary tumors. In 2022, Demarchi et al. showed that the canonical wnt pathway is involved in prolactin production and TMZ treatment in pituitary tumors. Anti-dopamine prolactinomas associated with normal pituitary undergo β-catenin relocalization. Furthermore, TMZ inhibits the tumorigenicity of prolactinomas by reducing the activation of β-catenin and the production of PRL (81). That same year, the team reported a case of an aggressive ACTH tumor in which TMZ resistance appeared relevant to β-catenin activation (82). TMZ is generally well tolerated and safe. The most common side effects are vomiting, nausea, fatigue, neutropenia, and thrombocytopenia. However, severe hematologic adverse events have also been reported, including aplastic anemia (83) and myelodysplastic syndrome (84). Drug rash, eosinophilia, systemic symptoms syndrome, and opportunistic infection (85) are the sporadic but highly challenging complications of TMZ therapy (86). The Wnt pathway is vital in treating prolactinoma, aggressive ACTHPA, and TMZ tumors, providing a new direction for curing aggressive and drug-resistant PAs.

Natural plant products have become an essential source of new anticancer drugs due to their multiple modes of action, multiple targets, safety, low toxicity, and few side effects (87). In 2017, Li et al. found that the CAG of Yunshi can induce apoptosis in cells of PAs by inhibiting the Wnt pathway. Moreover, dysregulation of the Wnt pathway may trigger ER stress in AtT-20 cells and play a vital role in apoptosis (88). In the same month, the research team found that tanshinone IIA increased β-catenin phosphorylation, inhibited β-catenin nuclear translocation, decreased β-catenin/TCF-4 complex formation, and TCF-LEF luciferase reporter gene activity, and subsequently decreased expression of cyclin D1 and MGMT. Ultimately, it can induce apoptosis in cells of PAs (89).

In addition to these drugs, it is also worth mentioning that they improve TME through the wnt pathway, thereby enhancing the efficacy of immunotherapy (90). The TME plays a constructive role in stimulating the invasiveness and persistence of PAs. It contains an extracellular matrix, CAFs, oncogenic immune cells, and other factors that can influence tumor tissue behavior. For example, TAM and TILs are related to NFPA, GH adenoma invasiveness, and tumor-aggressive behavior (91). TGFβ, FGF2, cytokines, chemokines, and growth factors released by CAFs may facilitate drug resistance, tumor fibrosis, and inflammation in PRL tumors and GH adenomas. Inhibition of Wnt signaling further suppressed the proliferation of dopamine-resistant PRL tumor cells. Invasion proteins secreted by the extracellular matrix of malignant tumors are relevant to increased angiogenesis (91–93). The TME is regulated by complex interactions of Wnt agonists, antagonists, and anti-antagonists. Therefore, improving the TME through modulation of the Wnt pathway is crucial in treating aggressive and refractory PA.

**Figure 2:**
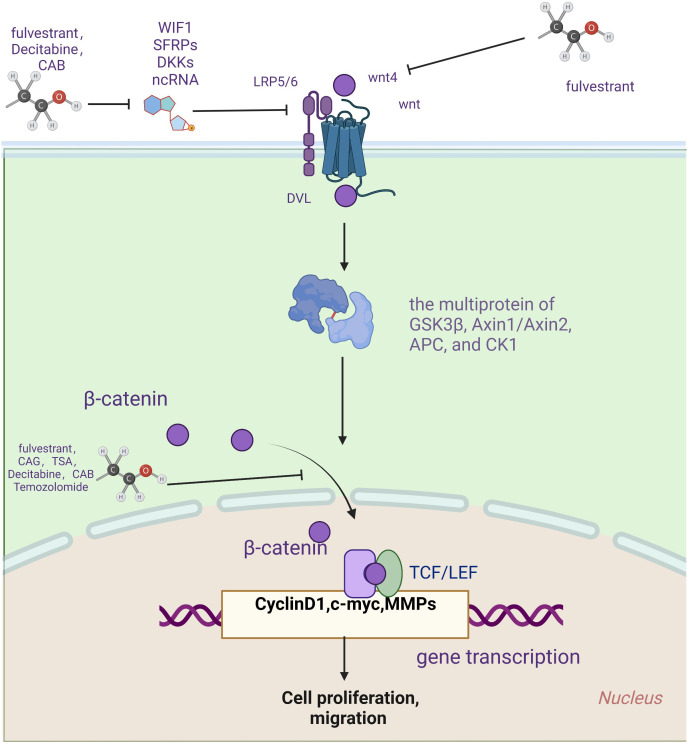
Drugs targeting wnt signaling pathway.

The Wnt signaling pathway has been proven to participate in various phases of regulating dendritic cells, the tumor immune cycle, T cells, and tumor cells. Although immunotherapy, especially immune checkpoint blockade therapy, has achieved good results in treating malignant tumors, some patients still have poor initial responses to immunotherapy or develop drug resistance after long-term treatment. Thus, inhibition of Wnt signaling plays a role in compensating for a deficiency in the immunotherapy of PA. Simultaneous use of Wnt pathway suppressants is supposed to improve TME and immunotherapy’s efficacy, pointing out a new direction for treating PA (**Table 2**; **Figure 2**).

**Table 2:** Related research on drugs targeting WNT signaling pathway.

drugs	years.	the targets of anticancer drugs	Adverse reaction	related findings.
fulvestrant	2012	Wnt4, WIF1 β-catenin, ERa	Vasodilation, nausea, Hepatotoxic (jaundice, elevated transaminases)	(69)
	2013			(70)
	2014			(71, 72)
Cassian-type diterpenes (CAG).	2017	β-catenin	**/**	(88)
Tanshinone IIA. (TSA)	2017	β-catenin	**/**	(89)
Decitabine	2019	WIF1, β-catenin	febrile neutropenia, thrombocytopenia, neutropenia	(57)
Cabergoline(CAB)	2021	WIF1, β-catenin	valvular heart disease, psychiatric disorders	(79)
Temozolomide	2022	β-catenin, Cyclin D1	drug rash, eosinophilia, systemic symptoms syndrome, aplastic anemi, opportunistic infections (pneumocystis pneumonia)	(81, 82)

## Discussion and conclusion

This review provides an overview of the use of Wnt signaling pathway inhibitors in treating PA. It also discusses the current understanding of the Wnt signaling pathway concerning PA and reviews studies on drugs targeting this pathway for treating PA. The Wnt pathway has traditionally been divided into canonical and atypical pathways, which form networks that communicate with other pathways and co-regulate interactions during the development of PA. Wnt signaling pathway Inhibitors include not only the SFRP family, WIF1, and DKK but also ncRNAs. ncRNAs have a dual role as inhibitors to suppress the ontogenesis of PA and as factors that spark Wnt signaling in the cells of PA.

Fulvestrant, a novel estrogen receptor antagonist, has been identified as a promising therapeutic agent for treating PA by targeting the Wnt pathway. It can treat PA by regulating the wnt pathway inhibitor. Drugs used to treat PA include fulvestrant, decitabine, TMZ, CAB, and natural compounds of plant origin, such as CAG and tanshinone IIA. They are closely related to the wnt signaling pathway. However, the specific mechanisms involved in wnt signaling remain unclear and deserve further experimental investigation. TME plays a vital role in the aggression and persistence of PA. Conventional immunotherapy cannot cover all types of PA treatment. Therefore, the combined use of Wnt modulators is expected to improve TME and immunotherapy’s efficacy, which points out a new direction for treating PA.

Future research regarding the wnt signaling pathway in PA should focus on (1) the Mechanism of the wnt signaling pathway in NFPA and prolactinomas. (2) achieving a deeper understanding of crosstalk among these pathways (e.g., Wnt, MAPK, TGF-b, Hippo, VEGF, JAK-STAT, PI3K-Akt, ErbB, TGF-b, and Ras, SOX2, Shh pathways); (3) Inhibitors of wnt signaling in PA should pay more attention to ncRNAs and to gain insight into their roles in wnt signaling in PA. (4) Targeting the wnt signaling pathway improves the TME in refractory PA. (5) Targeting the wnt pathway combined with other therapies covers more types of PAs.

In summary, the wnt signaling pathway plays a significant part in guiding the development and pathogenesis of PA. Abnormal expression of Wnt-signaling inhibitors may be related to the cell multiplication, invasion, and recurrence of PA. Currently, there are few studies on the wnt signaling pathway in PA. Furthermore, studies on these drugs have yet to go into sufficient depth into the mechanism of action. Finding new therapeutic targets and combining them with traditional treatments will reveal novel directions for the individualized treatment of PA, which needs further research.

## Author contributions

WW conceived of the topic of the review, performed the literature searches and wrote the manuscript. LM, YZ, ML, WY, and XL contributed to intellectual development and helped to edit the final manuscript. All authors contributed to the article and approved the submitted version.

## Glossary

**Table:**

PA	Pituitary adenoma
FZD	Frizzled
LRP5/6	the co-receptor low-density lipoprotein receptor-related protein 5/6
DVL/Dsh	Dishevelled
GSK3β	Glycogen synthase kinase 3β
APC	Adenomatous polyposis coli protein
CK1	Tyrosine kinase 1
LEF	Lymphatic enhancer-binding factor
TCF	T-cell factor
MMPs	Matrix metalloproteinases
PCP	Planar cell polarity
PLC	Phospholipase C
PKC	Protein kinase C
CaMkII	Calmodulin-sensitive protein kinase II
Calcineurin	Calcium-sensitive phosphatase calcium-regulated neuro phosphatase
NFAT	Nuclear factor of activated T cell
NFPA	Non-functioning pituitary adenoma
SFRP	secreted frizzled-associated protein
PITX2	pituitary homeobox 2
WIF1	Wnt Inhibitor 1
SLC20A1	the transcription of NF-κB-activated phosphate transporter 1
CDKN2A/p16	Cyclin-dependent kinase inhibitor 2A/p16
TGF-β	Transforming growth factor-β
NF-κB	Nuclear factor-κB
CS	Cushing’s syndrome
PRL	Prolactin
MITF	Malaysia international travel fair
NEK2	NIMA-associated kinase 2
CAB	cabergoline
TMZ	temozolomide
CAG	Cassian-type diterpene
MGMT	O6-Methyl guanine-DNA methyltransferase
TME	tumor microenvironment
CAFs	cancer-associated fibroblasts
TAMs	tumor-associated macrophages
TILs	tumor-infiltrating lymphocytes.
